# A standardized *Boswellia serrata* extract shows improvements in knee osteoarthritis within five days-a double-blind, randomized, three-arm, parallel-group, multi-center, placebo-controlled trial

**DOI:** 10.3389/fphar.2024.1428440

**Published:** 2024-07-18

**Authors:** Anju Majeed, Shaheen Majeed, G. Satish, R. Manjunatha, Shaikh Nawazish Rabbani, Neelanagowda V. P. Patil, Lakshmi Mundkur

**Affiliations:** ^**1**^ Sami-Sabinsa Group Limited, Peenya Industrial Area Bangalore, Bangalore, Karnataka, India; ^2^ Sabinsa Corporation, East Windsor, NJ, United States; ^3^ Nuha Hospital, Guntur, Andhra Pradesh, India; ^4^ KR Hospital, Department of Orthopedics, Mysore Medical College and Research Institute K R Hospital, Mysore, Karnataka, India

**Keywords:** *Boswellia serrata* extract, 3-acetyl-11-keto-β-boswellic acid, osteoarthritis, Boswellin Super, visual analog scale, WOMAC, lequesne functional index quality of life

## Abstract

**Background:**

Boswellin^®^ Super is a standardized extract of *Boswellia serrata* Roxb gum resin, standardized to contain 30% 3-acetyl-11-keto-β-boswellic acid along with other β-boswellic acids (BSE). A randomized, double-blind, placebo-controlled clinical trial was conducted at two doses of BSE to understand its safety and efficacy in supporting joint health and improving mobility and symptoms of osteoarthritis (OA) of the knee.

**Methods:**

Based on the inclusion/exclusion criteria, 105 newly diagnosed participants with degenerative hypertrophy OA were recruited and randomized into Placebo, BSE-150 mg or BSE-300 mg (n = 35 in each group) to receive either 150 mg or 300 mg BSE or a placebo tablet twice a day for 90 days. All the participants were evaluated for pain and physical function using the standard tools including the Visual Analog Scale (VAS), Western Ontario and McMaster Universities Osteoarthritis Index (WOMAC), Lequesne Functional Index (LFI), EuroQol- 5 Dimension (EQ-5D) quality of life, 6-min walk test at day 0, days 5, 30, 60 and 90 of treatment. Additionally, the circulating levels of inflammatory biomarkers, tumor necrosis factor-α (TNFα), high-sensitive C-reactive protein (hs-CRP), and interleukin-6 (IL-6) were evaluated. Safety was evaluated by blood biochemical, hematological analysis, urinary analyses and by monitoring adverse events throughout the study.

**Results:**

Ninety-eight subjects completed the study. Improvements in pain scores were observed as early as 5 days after the start of the supplement in the BSE-150 and BSE-300 groups. By 90 days, the VAS pain score reduced by 45.3% and 61.9%, WOMAC- total score improved by 68.5% and 73.6% in the BSE-150 and BSE-300 groups respectively. WOMAC pain (70.2%, 73.9%, WOMAC stiffness (65.6%,68.9%), WOMAC function (68.8%,74.2%), LFI severity (50%,53.3%), decreased and EQ5D (56.9%, 62.9%) and distance walked in 6 minutes (21.2%, 21.9%) improved in the BSE-150 and BSE-300 groups in 90 days. Further, the levels of TNFα, hs-CRP, and IL-6 were found to decrease in the serum in BSE-supplemented participants. No significant adverse events were recorded during the study.

**Conclusion:**

The study confirms that **Boswellin® Super** can be used as a safe and effective supplement to support joint health and mobility in the management of osteoarthritis.

**Clinical Trial Registration:**

https://ctri.nic.in/Clinicaltrials/pmaindet2.php?EncHid=NzU2Nzc=&Enc=&userName=CTRI, identifier CTRI/2022/11/047397

## 1 Introduction

Osteoarthritis (OA) is the most prevalent form of joint inflammatory condition in adults leading to chronic pain and loss of mobility (Glyn-Jones et al., 2015). According to World Health Organization (Vos et al., 2019) data, 528 million individuals worldwide suffer from osteoarthritis (OA), with 73% of those affected being older than 55 years (2019, 2019). Apart from age, genetics, obesity, joint injury, gender, and occupational activities are other risk factors for OA. The quality of life of millions of individuals can be improved for decades by reducing the consequences of OA (Lau et al., 2000; Snoeker et al., 2020).

Although OA affects all joints, it is most common in knee and hip joints. It is characterized by the degradation of articular cartilage leading to fibrosis, joint degeneration, and damage to the entire articular surface (Felson, 2004). The primary focus of OA management is to reduce pain using nonsteroidal anti-inflammatory drugs (NSAIDs) and specific cyclooxygenase II (COX-2) inhibitors (Wright, 2002). These drugs are known to be associated with gastrointestinal, renal, and cardiovascular risks (Singh, 1998; Harirforoosh et al., 2013). Thus, there is a need for an effective alternate therapy that can reduce the use of these drugs or complement their use with minimal adverse side effects.

Boswellic acids (BAs), the triterpenes present in the gum resins of *B. serrata* Roxb. (Family: Burseraceae) have been traditionally used in the Ayurvedic system of medicine as an antioxidant and anti-inflammatory agent to manage diseases such as rheumatoid arthritis, chronic bronchitis, asthma and chronic inflammatory bowel diseases and OA (Basch et al., 2004; Ahmed et al., 2013; Majeed et al., 2020a). The β-pentacyclic triterpene acids in *Boswellia serrata* including 3-acetyl-11-keto-β-boswellic acid (AKBBA), 11-keto-β-boswellic acid (KBBA), β-boswellic acid (BBA), and 3-acetyl-β-boswellic acid (ABBA), represent the major bioactive boswellic acids in the gum resin (Büchele et al., 2003; Poeckel and Werz, 2006). Amongst these boswellic acids, AKBBA was found to be a potent inhibitor of leukotriene-mediated inflammatory pathways and 5-lipoxygenases (5-LO) activities (Sailer et al., 1996; Schweizer et al., 2000). It has been shown to inhibit inflammatory mediators, matrix metalloproteins, and other adhesion factors in *in vitro* studies (Roy et al., 2005; Syrovets et al., 2005; Roy et al., 2006; Majeed et al., 2021). In a meta-analysis including seven clinical trials involving 545 patients, Boswellia and its extract were reported to have a positive effect on relieving pain, and stiffness and improving joint function (Yu et al., 2020). We have earlier reported the beneficial effect of Boswellin^®^ Super, a standardized extract of *B. serrata* containing not less than 30% 3-acetyl-11-keto-β-boswellic acid along with other β-boswellic acids, *in* an experimental collagen-induced preclinical arthritis model (Majeed et al., 2021). The preclinical safety of this compound has also been established as per the regulatory requirements (Majeed et al., 2020b). In the present study, we evaluated the clinical effects of Boswellin^®^ Super in a randomized double-blind placebo-controlled study as a standalone supplement in individuals with mild to moderate degenerative hypertrophy osteoarthritis.

## 2 Materials and methods

### 2.1 Materials

Boswellin^®^ Super is a standardized extract of *B. serrata* containing not less than 30% 3-acetyl-11-keto- β-boswellic acid (AKBBA), 7.5% β-boswellic acid, 3.5% of 3-O-acetyl-β boswellic acid, and 1.5% 11-Keto-β-boswellic acid as analyzed by HPLC. The content of the total identified beta boswellic acids was between 50%–55% in the extract. BBA and ABBA are detected at 210 nm and KBBA and AKBBA at 254 nm. The HPLC chromatograms are shown in the Supplementary Figures S1A, B. Boswellin^®^ Super, henceforth termed (BSE), was formulated into tablets containing either 150 mg or 300 mg of BSE in two doses. Placebo capsules contained 300 mg of microcrystalline cellulose. All three tablets were of the same size, color, and weight. The standardized Boswellin^®^ Super FJ was provided by Sami-Sabinsa Group Limited.

### 2.2 Study design

A double-blind, randomized, three-arm, parallel-group, multi-center, placebo-controlled trial was conducted in 105 individuals for 90 days. The study was conducted in two sites, Nuha Hospital, Guntur, and Mysore Medical College, and Research Institute, Mysore, from 5 January 2023 to 3 November 2023. The study protocol, CW/111/BSEE_OSAR/II/AUG/22 was reviewed and approved by the institutional Ethics committee of Nuha Hospital on 8 November 2022 and Mysore Medical College and Research Institute on 24 January 2023. Good Clinical Practice as required by the International Conference on Harmonization was followed while conducting the study. The trial was registered prospectively with the Clinical Trial Registry of India (CTRI) with the registration number CTRI/2022/11/047,397 [Registered on: 17/11/2022].

### 2.3 Sample size

Based on an earlier publication on B serrata extract, using 80% power and alpha = 0.05 significance level assuming a correlation of 0.2, the required total sample size was calculated as 90 for evaluation. Allowing for a 15% drop-out rate, the required sample size for recruitment was fixed at 105 in a 1:1:1 ratio between three arms. (Majeed et al., 2019). The details of the calculations are given in the supplementary section.

### 2.4 Study population

Male and female participants in the age group of 40–75 years, newly diagnosed with degenerative hypertrophy OA were enrolled in the study.

#### 2.4.1 Inclusion criteria

Individuals with degenerative hypertrophy OA with Kellgren-Lawrence (KL) grades I-II based on an X-ray of the knee joint and anteroposterior view on standing were enrolled in the study. The participants had pain perception ranging from 30 to 100 in the Visual Analog Scale (VAS). Other inclusion criteria were willingness to comply with the study protocol and attend regular follow-up visits. All the participants signed the written informed consent form.

#### 2.4.2 Exclusion criteria

The participants were excluded if they had nondegenerative joint diseases that can interfere with the evaluation of OA (Rheumatoid arthritis, active gout, recent joint trauma, or joint infection), KL grade of III or higher, incapacitated or bound to wheelchair or bed and unable to carry out self-care activities and those with a history of knee or hip replacement surgery. Participants with prior treatment with corticosteroids, glucosamine, chondroitin, hyaluronate, glucocorticoids, NSAID or steroids, and herbal or alternate medicines within 1 month before screening, were also excluded from the study. Other exclusion criteria were the presence of chronic diseases and hypersensitivity to herbal extracts or dietary supplements. Detailed exclusion criteria are given in the supplementary section.

### 2.5 Randomization and blinding

The randomization sequence was prepared by an independent statistician, independent of the sponsoring organization and the investigators. Subjects were randomized using a predetermined block randomization schedule generated using computer-based randomization software (SAS 9.3). The study followed a double-blind design, wherein neither the investigator nor the subjects were aware of the treatment assignment. The principal investigator used randomly generated alphanumeric codes to refer to each of the investigational products. The randomization codes were kept strictly confidential and were accessible only to authorized persons on an emergency basis as per the standard operating procedures until the time of unblinding.

The investigational products (IP) of all three arms were manufactured, packaged, and stored in identical bottles to ensure proper blinding such that neither the investigator nor the subjects would be able to identify the dispensed IP to be an active or a placebo. All three tablets were coated with the same color to mask the identity and smell of the product. The subjects were trained to self-administer the IP at their home during the study period. They were asked not to open the IP bottles or discuss the color, nature, odor, or any description of the product. Further, the products were labeled using an alphanumeric number to mask the identity of the product.

### 2.6 Intervention and measurements

The study participants were instructed to consume one tablet of either BSE- 150 mg, BSE 300 mg, or a matching placebo (300 mg microcrystalline cellulose) twice a day after breakfast and dinner for 90 days. All the tablets were of the same size, weight, and color and comparable to each other. Compliance was assessed by recording the number of tablets dispensed to the subject and the numbers returned at each visit in the case record form.

Participants visited the study site on Screening (day −5), baseline visit (day 0), day 5, day 30, day 60, and day 90. Follow-up was conducted 15 days after the last visit (Day 105).

WOMAC, VAS Pain Scale, European Q5D QOL, Lequesne functional Index tests, Six Minute Walk Test, and Physician Global Assessment were administered on the baseline, and days 30, 60, and 90. X-ray of the knee (Anteroposterior view), and biomarkers, were analyzed on baseline and day 90. Safety assessments were carried out on day 0, day 30, and day 90. The participants are allowed to take Celecoxib 200 mg orally a day as a rescue medication if required. Residual efficacy was evaluated 15 days after the end of the study period by asking the participant about his/her perception of pain and recording the VAS score.

### 2.7 Outcomes

The Primary endpoints of the study were the mean change in the total scores in modified WOMAC and VAS from day 0 to day 30, day 60, and day 90. The secondary endpoints included the mean change in WOMAC- pain, stiffness and function subscales, mean changes in distance walked in 6 min, European Q5D Quality of Life, Lequesne Functional Index and physician’s global assessment from day 0 to day 30, day 60 and day 90, and the mean change in hs-CRP, ESR, TNF-α, and IL-6 levels in serum, and changes in the radiological parameters from day 0 and day 90. Safety was assessed through laboratory tests, changes in vital signs, physical examination, and adverse events from day 0 to day 30, day 60, and day 90.

#### 2.7.1 Kellgren-Lawrence grading

The presence of OA was assessed based on the X-ray of the knee joint, AP view on standing as Grade 0-Normal joint with radiological findings in OA; Grade I-Doubtful narrowing of joint space and possible osteophytic lipping; Grade II- Definite osteophytes and possible narrowing of joint space: Grade III-definite narrowing of joint space, with moderate multiple osteophytes, and, possible deformity of bone contour; Grade IV-Marked narrowing of joint space with large osteophytes, definite deformity of bone contour and severe sclerosis (Kohn et al., 2016).

#### 2.7.2 Visual analog scale (VAS)

The pain VAS is a unidimensional measure of pain intensity and is considered a reliable and valid tool for capturing the pain intensity in subjects with chronic pain (Myles et al., 2017). Using a ruler, the score is determined by measuring the distance (mm) on the 10-cm line between no pain (0) to extreme pain (10), providing a range of scores from 0 to 100. A higher score indicates greater pain intensity. Subjects were asked to report pain intensity “in the last 48 h” or an average pain intensity.

#### 2.7.3 Modified Western Ontario and McMaster universities arthritis index score (WOMAC)

The modified WOMAC score (CRD- Pune version) consisted of 27 questions in three different categories (Arun Kumar M, 2021). Pain had five questions with a score range of 0–20; Stiffness had two questions with a range of 0–8, physical function had 17 questions with a score range of 0–68. Each question has five responses starting from none, mild, moderate, severe, and extreme: 0–four on the Likert scale. The scores for each subscale were added to get the total score of 96. Higher scores were associated with increased severity.

#### 2.7.4 Physician Global Assessment

The Physician Global Assessment (PGA) of treatment response is assessed by the physician and measures the overall response to treatment. Based on physical examination, medical history, assessment of objectives of treatment, and patient interviews, the physician records a score ranging from −4 indicating worsening, and +4 indicating improvement.

#### 2.7.5 Six-minute walk test

Six-minute walking test was performed in a long, straight hospital corridor, over a distance (ATS Committee on Proficiency Standards for Clinical Pulmonary Function Laboratories, 2002). Each participant was asked to walk back and forth along the corridor as briskly as possible. The number of laps and the total distance walked in 6 min were calculated.

#### 2.7.6 Lequesne Functional Index (LFI)

The Lequesne functional index is a knee OA-specific questionnaire, which is used for assessing the prognosis (Lequesne, 1997). The questionnaire covers pain or discomfort, and activities of daily living. Each section carries a score of 0–8. The total scores are added together to get the functional index score of 24 as the maximum. A score of 0 indicates no disease/handicap, 1–4: Mild Handicap, 5–7: Moderate, 8–10: Severe, 11–13: Very severe, >14: Extremely severe.

#### 2.7.7 EuroQol- 5 dimension (EQ-5D)

EQ5D is a generic questionnaire to measure health-related quality of life. The self-assessment questionnaire is a description of the subject’s current health w. r.t mobility, self-care, usual activities, pain/discomfort, and anxiety/depression. The answers are converted into the EQ-5D index, wherein a value of 0 indicates poor health and one for perfect health. The questionnaire also includes a Visual Analog Scale, to grade the perceived health status from 0 (the worst possible health status) to 100 (the best possible health status) (Balestroni and Bertolotti, 2012).

#### 2.7.8 Biochemical analysis

Fasting blood samples (10 mL) were collected from each participant, via venipuncture with a Vacutainer system (Becton Dickinson Biosciences, NJ, USA) by trained laboratory staff (EDTA-treated tubes for hematology and untreated tubes for biochemistry tests). Hematological parameters were measured by Sysmex XN1000 (Sysmex Corporation, Kobe, Japan); and biochemical parameters, including hs-CRP by Cobas 400 (Roche Diagnostics, Mannheim, Germany) per manufacturer’s instructions. Biomarkers (TNF-α and IL-6) were measured using commercial ELISA kits (Diaclone SAS, France) as per the manufacturer’s instructions.

### 2.8 Statistical analysis

The statistical analysis was carried out by an independent statistician, not associated with the sponsor or the investigator. All the subjects who completed the study were considered for data analysis. The normality of distribution was tested by a one-sample Shapiro-Wilk normality test for continuous variables. Based on the normality, the quantitative variables were described either as mean and standard deviation or median and interquartile range. Repeated measures of one-way ANOVA followed by Dunnett’s *post hoc* test were performed within the group comparison of parameters at every visit. The primary and secondary efficacy endpoints were compared between the groups by Two-way ANOVA followed by Turkey’s *post hoc* test. The chi-square test was used to compare the categorical variables which were presented as frequency and percentage of the population. The change in quantitative variables from baseline at different time points was compared to differentiate the treatment effect between the treatment groups. The level of statistical significance is defined as *p* < 0.05.

## 3 Results

### 3.1 Demographic and baseline characteristics

**FIGURE 1:**
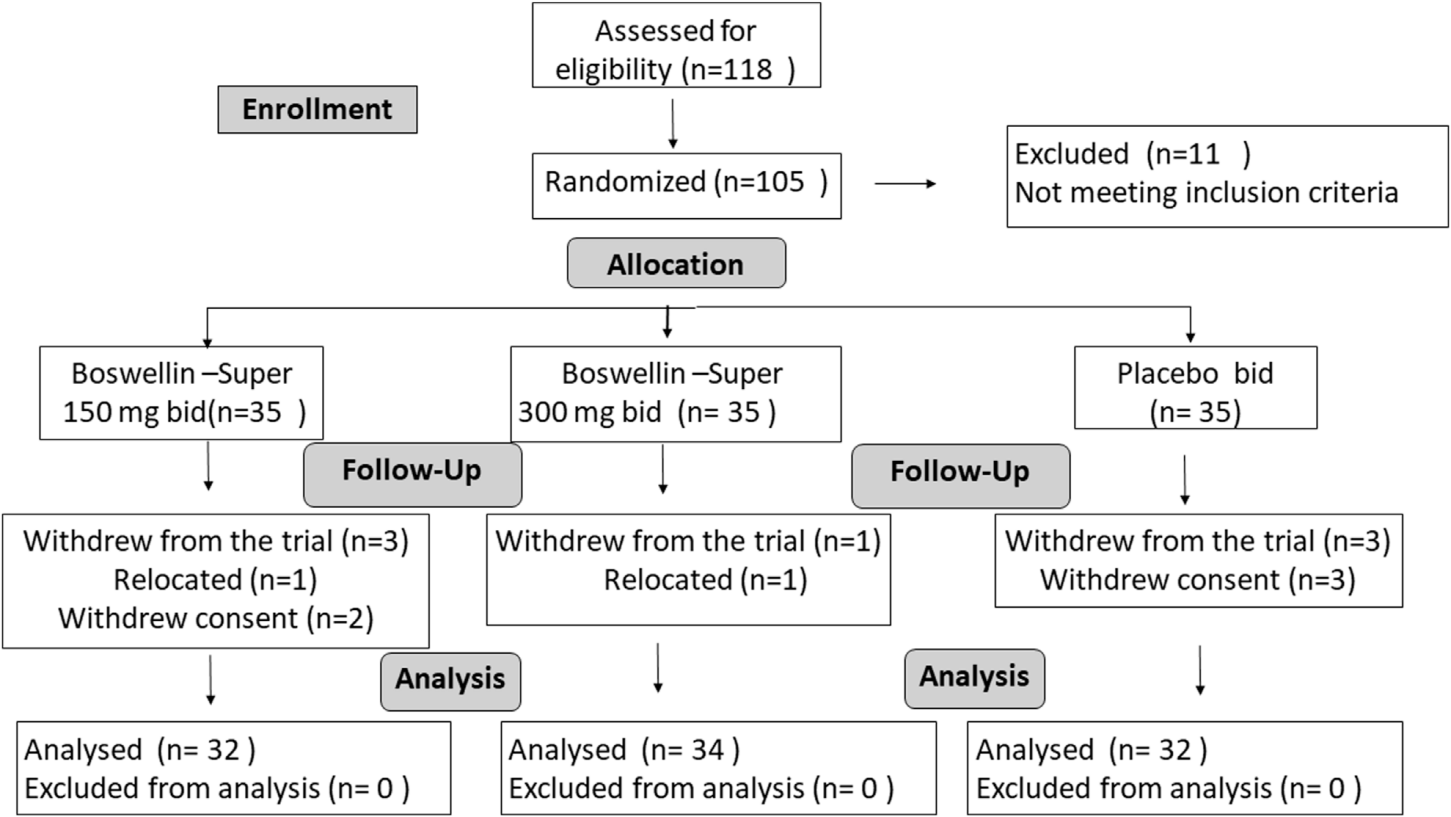
Consort Diagram: Consort flow chart showing the enrolment, allocation, follow-up, and analysis of participants.

A total of 118 subjects were screened, and 11 were screen failures. Two subjects withdrew from the study before randomization and 105 eligible subjects were randomized to the BSE-150, BSE-300, and placebo groups, with n = 35 in each group (Figure 1). Seven subjects withdrew during the trial, within 5 days of the start of the trial, two participants relocated and could not continue, while five withdrew their consent. Finally, 98 subjects completed the study, n = 32 in BSE-150 (17 males and 15 females) and placebo (7 males and 25 females) and N = 34 in BSE-300 (9 males and 25 females). The mean age of participants was 52.2 ± 7.2 in placebo, 54.7 ± 7.9 in BSE-150, and 51.8 ± 5.7 in BSE-300. None of the participants were taking any medication for pain at the time of enrollment as per the exclusion criteria. The demographic characteristics are provided in Table 1.

**TABLE 1:** Demographic details.

Parameter		Placebo	BSE-150	BSE-300	*p*-value
Age		52.22 ± 7.23	54.69 ± 7.94	51.79 ± 5.76	0.197
Gender	Male	9	18	9	0.03
	Female	26	17	26	
Height (cm)		157.11 ± 6.55	158.15 ± 6.87	157.31 ± 6.9	0.792
Weight (kg)		65.57 ± 6.31	65.94 ± 6.18	65.25 ± 6.11	0.896
BMI (kg/m^2^)		26.29 ± 2.56	26.34 ± 2.69	26.33 ± 2.1	0.996
Systolic BP (mmHg)		123.29 ± 3.31	122.83 ± 5.11	122.2 ± 4.62	0.588
Diastolic BP (mmHg)		76.34 ± 8.4	76.54 ± 8.91	75.63 ± 12.09	0.921
Body Temperature (°F)		97.81 ± 1.06	98.11 ± 0.7	97.8 ± 1.18	0.336
Respiratory Rate (breaths/min)		16.69 ± 0.96	16.74 ± 1.07	16.49 ± 1.12	0.564
Smoking		No	No	No	
Alcohol abuse		No	No	No	
Ethnicity		Asian	Asian	Asian	
VAS score		43.75 ± 7.07	46.25 ± 7.12	49.41 ± 6.96	0.03

Baseline demographics were compared by One-way ANOVA., Mean and Standard deviation are presented. BMI: body mass index, BP: blood pressure, VAS: visual analog scale for pain.

### 3.2 Effect of BSE on VAS and WOMAC scores

**FIGURE 2:**
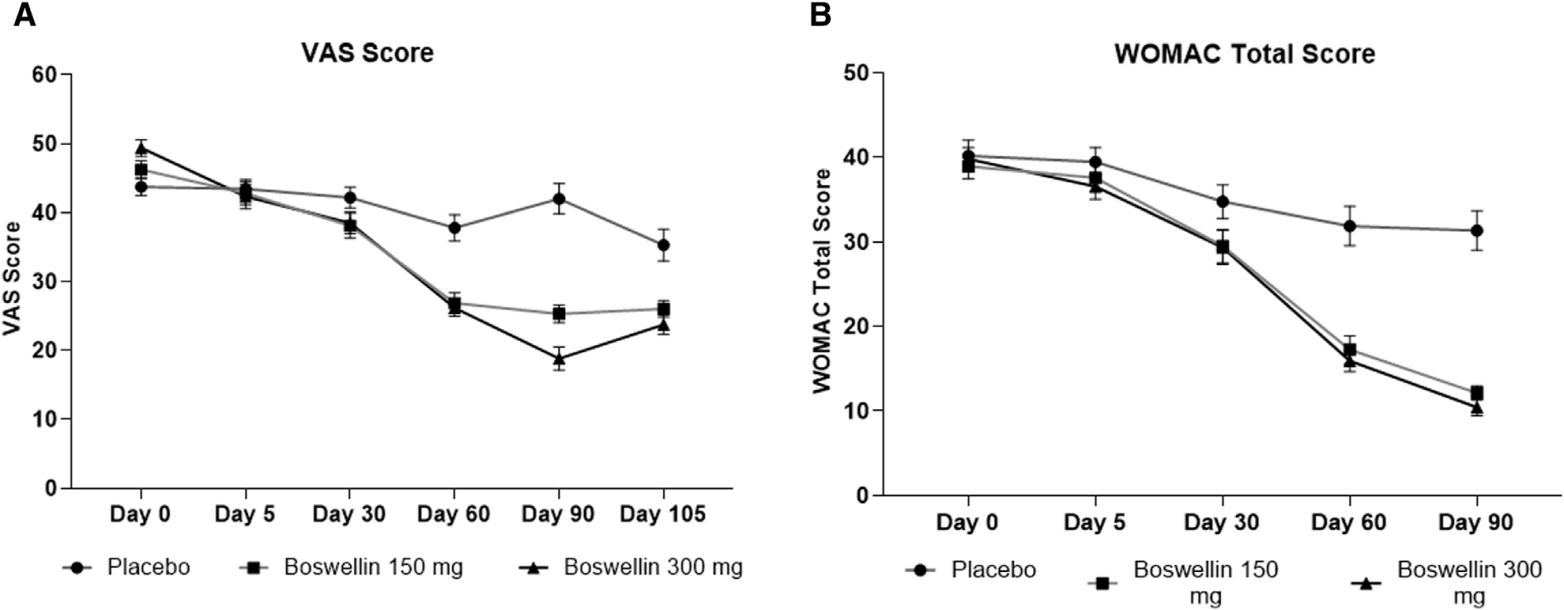
VAS and WOMAC total scores **(A)**: Visual analog scale for pain (VAS), **(B)**: Western Ontario McMaster Index (WOMAC) total scores at different time points day 0, day 30, day 60 and day 90 in placebo, BSE-150 and BSE-300 groups. (Day 105 included for VAS scores). Each point represents Mean ± SEM. The decrease in VAS score was significant (*p* = 0.002) on day 60 in comparison to day 0 in placebo, and from day 30 to 105 compared to day 0 (*p* < 0.001) in BSE-150 and BSE-300 compared to day 0.

**FIGURE 3:**
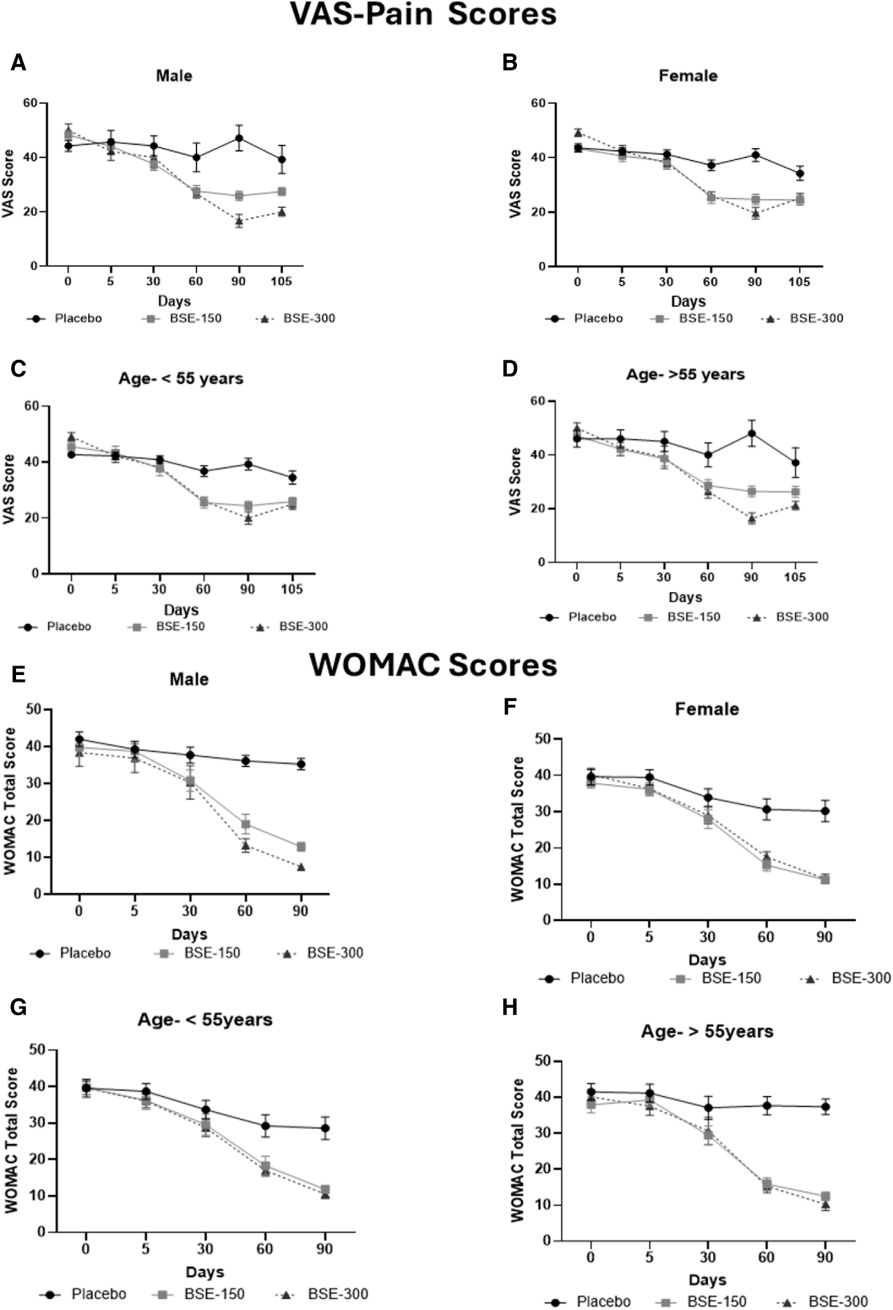
VAS and WOMAC total scores in age and gender specific groups: Visual analog scale for pain (VAS) in **(A)**: Male, **(B)**: Female, **(C)**: Age <55 years, **(D)**: > 55 years, Western Ontario McMaster Index (WOMAC) total scores in **(E)**: Male, **(F)**: Female, **(G)**: Age < 55 years, **(H)**: > 55 years in a gender and age specific analysis, in placebo, BSE-150 and BSE-300 groups. Each point represents Mean ± SEM. Study participants were grouped into two age groups <55 years (40–55 years) and >55 years (56–70 years). The parameters are analyzed in male and female participants and in the two age groups.

The visual analog score of pain reduced gradually from day 0 to day 90 in all groups, although the magnitude of the drop was considerably greater in the BSE-150 and BSE-300 groups than in placebo (Figure 2). The mean VAS pain scores decreased from 43.7 ± 7.1 to 43.4 ± 7.8 42.2 ± 8.7, 37.8 ± 10.7 and 42.0 ± 12.3 on days 5, 30,60 and 90 in the placebo group, while the decrease in VAS was consistent inBSE-150 (46.2 ± 1.3 to 42.8 ± 1.7, 38.1 ± 1.8, 26.8 ± 1.5, 25.3 ± 1.3) and BSE-300 (49.4 ± 1.2 to 42.3 ± 1.8, 38.5 ± 1.6, 26.2 ± 1.2 and 18.8 ± 1.7) on days 5, 30,60 and 90. The pain levels remained reduced in the BSE-150 and BSE-300 groups (26.1 ± 1.2 and 21.8 ± 1.5), even after 15 days of study completion. The two-way RMANOVA test for the interaction between groups and the number of days of treatment was significant (*p* < 0.001). Turkey’s multiple comparisons revealed a significant difference between placebo and BSE-150 and BSE-300 on days 60 and 90 (<0.0001 for both time points) (Table 2). The gender distribution was not uniform in the three groups, as the number of female participants was higher in placebo and BSE-300. A gender-specific analysis demonstrated a significant improvement in VAS scores after 90 days in men and 60 days in women (Figure 3; Table 3). Two-thirds of the participants were less than 55 years of age and showed a significant improvement in VAS scores in BSE-supplemented groups after 60 days, while a significant improvement was observed in older (>55 years) participants by 90 days (Figure 3; Table 3).

**TABLE 2:** Comparison of mean VAS and WOMAC Scores between the groups by Two-way ANOVA.

Parameter	VAS			WOMAC TS		
	Placebo vs BSE-150	Placebo vs BSE-300	BSE150 vs BSE-300	Placebo vs BSE-150	Placebo vs BSE-300	BSE-150 vs BSE-300
Day 0	−2.50 (−6.74 o1.74) 0.33	−5.66 (−9.80 to −1.52) 0.0047	−3.16 ( −7.30 to 0.98) 0.167	1.25 ( −4.37–6.87) 0.854	0.39 ( −5.12–5.91) 0.983	−0.86 (−5.62 to 3.91) 0.902
Day 5	0.63 (−4.64–5.89) 0.956	1.09 (−4.37–6.54) 0.882	0.46 (−5.46–6.38) 0.981	1.91 (−3.71–7.53) 0.695	2.94 (−2.44–8.32) 0.394	1.03 (−4.27–6.34) 0.887
Day 30	4.06 (−1.66–9.79) 0.212	3.66 (−1.64–8.96) 0.230	−0.40 (−6.20 to 5.39) 0.984	5.25 (−1.42–11.92) 0.152	5.40 (−1.41–12.21) 0.146	0.15 (−6.62–6.92) 0.998
Day 60	10.94 (5.11–16.77) <0.001	11.64 (6.24–17.03) <0.001	0.70 (−3.95–5.34) 0.930	14.63 (7.81–21.44) <0.001	15.93 (9.54–22.33) <0.001	1.31 (−3.64–6.26) 0.801
Day 90	16.72 (10.64–22.80) <0.001	23.21 (16.61–29.81) <0.001	6.49 (1.44–11.54) 0.008	19.225 (13.19–25.25) <0.001	20.90 (14.74–27.07) <0.001	1.68 (−1.39–4.76) 0.392
Day 105	9.25 (2.92–15.58) 0.0026	11.55 (4.97–18.12) 0.0003	4.18 (−2.17–6.77) 0.438	ND	ND	ND
TWO WAY ANOVA						
Days	F (2, 95) = 13.25, *p* < 0.001			F (2, 95) = 14.20, *p* < 0.001		
Groups	F (3.62, 344.2) = 121.8, *p* < 0.001			F (2.61, 248.6) = 205.6 *p* < 0.001		
Interaction	F (10, 475) = 17.60 *p* < 0.001			F (8, 380) = 17.72 *p* < 0.001		

The difference in Mean value between the groups and the 95% of the difference are presented. Two-wayANOVA, was computed with groups and time as factors. F distribution (the distribution of the ratio of two estimates of variance), degrees of freedom numerator (dfn) and degrees of freedom denominator (dfd) are given. Post hoc analysis was carried out by Turkey’s multiple comparisons between placebo and BSE-150, and BSE-300. *p* < 0.05 was considered significant. VAS: visual analog scale for pain, WOMAC: Western Ontario McMaster Index.

**TABLE 3:** Impact of gender and age in mean change of VAS and WOMAC- Total scores at different time points.

Parameter	Mean (95% CI) of difference in mean between the groups										TWO way ANOVA		
	Day 0		Day 5		Day 30		Day 60		Day 90		Days	Group	Interaction
	Placebo vs BSE-150	Placebo vs BSE-300	Placebo vs BSE-150	Placebo vs BSE-300	Placebo vs BSE-150	Placebo vs BSE-300	Placebo vs BSE-150	Placebo vs BSE-300	Placebo vs BSE-150	Placebo vs BSE-300			
VAS Male	−3.95 (−10.90 to 3.003)	−5.71 (−13.84 to 2.411)	1.60 (−11.99–15.18)	3.49 (−10.86–17.84)	6.64 (−5.150–18.43)	4.29 (−10.12–18.69)	12.35 (−4.073–28.78)	13.33 (−3.060–29.73)	21.26 (6.711–35.81)	30.48 (15.68–45.27)	F (2, 30) = 6.023	F (3.302, 99.06) = 38.76	F (10, 150) = 7.470
*p*-value	0.331	0.193	0.946	0.796	0.320	0.722	0.140	0.107	0.008	0.0007	0.0063	<0.0001	<0.0001
VAS Female	0.27 (−4.539–5.072)	−5.60 (−10.60 to −0.6035)	1.73 (−4.368–7.835)	0.00 (−6.217 to 6.217)	2.53 (−5.848–10.92)	3.20 (−2.441–8.841)	11.87 (4.727–19.01)	11.20 (5.180–17.22)	16.33 (8.875–23.79)	21.40 (13.69–29.11)	F (2, 62) = 8.726	F (3.593, 222.7) = 70.06	F (10, 310) = 11.43
*p*-value	0.990	0.025	0.762	>0.9999	0.733	0.363	0.0008	0.0001	<0.0001	<0.0001	0.0005	<0.0001	<0.0001
VAS <55	−2.83 (−7.828 to 2.171)	−6.40 (−11.09 to −1.715)	−1.06 (−7.897 to 5.776)	0.10 (−6.294–6.491)	3.13 (−4.317–10.58)	2.65 (−2.186–7.483)	11.26 (4.489–18.04)	10.73 (5.013–16.45)	14.87 (8.362–21.39)	19.32 (11.84–26.80)	F (2, 60) = 8.163	F (3.755, 225.3) = 74.91	F (10, 300) = 9.839
*p*-value	0.358	0.005	0.922	0.999	0.557	0.387	0.0007	0.0001	<0.0001	<0.0001	0.0007	<0.0001	<0.0001
VAS >55	−1.14 (−10.49 to 8.200)	−4.00 (−13.34 to 5.336)	3.86 (−6.787–14.50)	3.27 (−8.342–14.89)	6.43 (−5.151–18.01)	5.91 (−8.257–20.08)	11.43 (−1.771–24.63)	13.64 (0.3072–26.97)	21.57 (7.462–35.68)	31.64 (17.49–45.78)	F (2, 32) = 5.796	F (3.007, 96.22) = 44.97	F (10, 160) = 8.270
*p*-value	0.947	0.522	0.630	0.756	0.351	0.550	0.094	0.045	0.004	0.0002	0.0071	<0.0001	<0.0001
WOMAC TS- Male	2.18 (−5.787–10.14)	3.56 (−7.733–14.84)	0.52 (−8.251–9.293)	2.40 (−9.531–14.33)	6.89 (−2.203–15.98)	7.38 (−6.157–20.92)	17.14 (9.467–24.82)	22.92 (16.74–29.10)	22.40 (17.20–27.60)	27.84 (22.87–32.81)	F (2, 30) = 6.367	F (3.150, 94.51) = 68.58	F (8, 120) = 8.178
*p*-value	0.772	0.685	0.988	0.856	0.161	0.342	<0.0001	<0.0001	<0.0001	<0.0001	0.0050	<0.0001	<0.0001
WOMAC TS (Female)	1.75 (−4.760–8.254)	−0.60 (−7.073 to 5.873)	3.32 (−3.414–10.05)	3.12 (−3.126–9.366)	5.92 (−2.868–14.71)	4.92 (−3.195–13.04)	15.41 (7.250–23.58)	13.12 (5.171–21.07)	18.97 (11.29–26.65)	18.72 (10.89–26.55)	F (2, 62) = 8.786	F (2.353, 145.9) = 119.5	F (8, 248) = 9.993
*p*-value	0.790	0.972	0.459	0.453	0.239	0.316	0.0001	0.0008	<0.0001	<0.0001	0.0004	<0.0001	<0.0001
WOMAC -TS <55	−0.13 (−7.791 to 7.528)	−0.06 (−7.506 to 7.384)	2.46 (−5.664–10.58)	2.64 (−4.305–9.581)	4.13 (−5.150–13.40)	4.94 (−3.513–13.40)	10.89 (1.221–20.57)	12.27 (3.848–20.69)	16.76 (8.564–24.95)	18.07 (9.802–26.34)	F (2, 60) = 5.299	F (2.517, 151.0) = 123.7	F (8, 240) = 7.793
*p*-value	0.999	1.000	0.741	0.629	0.528	0.340	0.024	0.003	<0.0001	<0.0001	0.0076	<0.0001	<0.0001
WOMAC TS > 55	3.57 (−4.635–11.78)	1.41 (−6.591–9.409)	1.91 (−6.031–9.860)	3.66 (−5.485–12.79)	7.67 (−2.873–18.22)	6.46 (6.275–19.20)	21.84 (13.98–29.71)	22.43 (14.54–30.32)	24.90 (18.37–31.43)	27.13 (19.99–34.27)	F (2, 32) = 14.81	F (2.536, 81.15) = 80.72	F (8, 128) = 12.70
*p*-value	0.526	0.896	0.814	0.576	0.182	0.418	<0.0001	<0.0001	<0.0001	<0.0001	<0.0001	<0.0001	<0.0001

The difference in Mean value between the groups and the 95% of the difference are presented. Two-way ANOVA, was computed with groups and time as factors Post hoc analysis was carried out by Turkey’s multiple comparisons between placebo and BSE-150, and BSE-300, and the *p* values are given for this comparison at different days. F distribution (the distribution of the ratio of two estimates of variance), degrees of freedom numerator (dfn) and degrees of freedom denominator (dfd) are given. Two-way ANOVA *p* values for days, group and their interactions are represented in the last three columns. Study participants were grouped into two age groups <55 years (40–55 years) and >55 years (56–70 years). The parameters are analyzed in male and female participants and in the two age groups. *p* < 0.05 was considered significant. VAS: visual analog scale for pain, WOMAC: Western Ontario McMaster Index.

**FIGURE 4:**
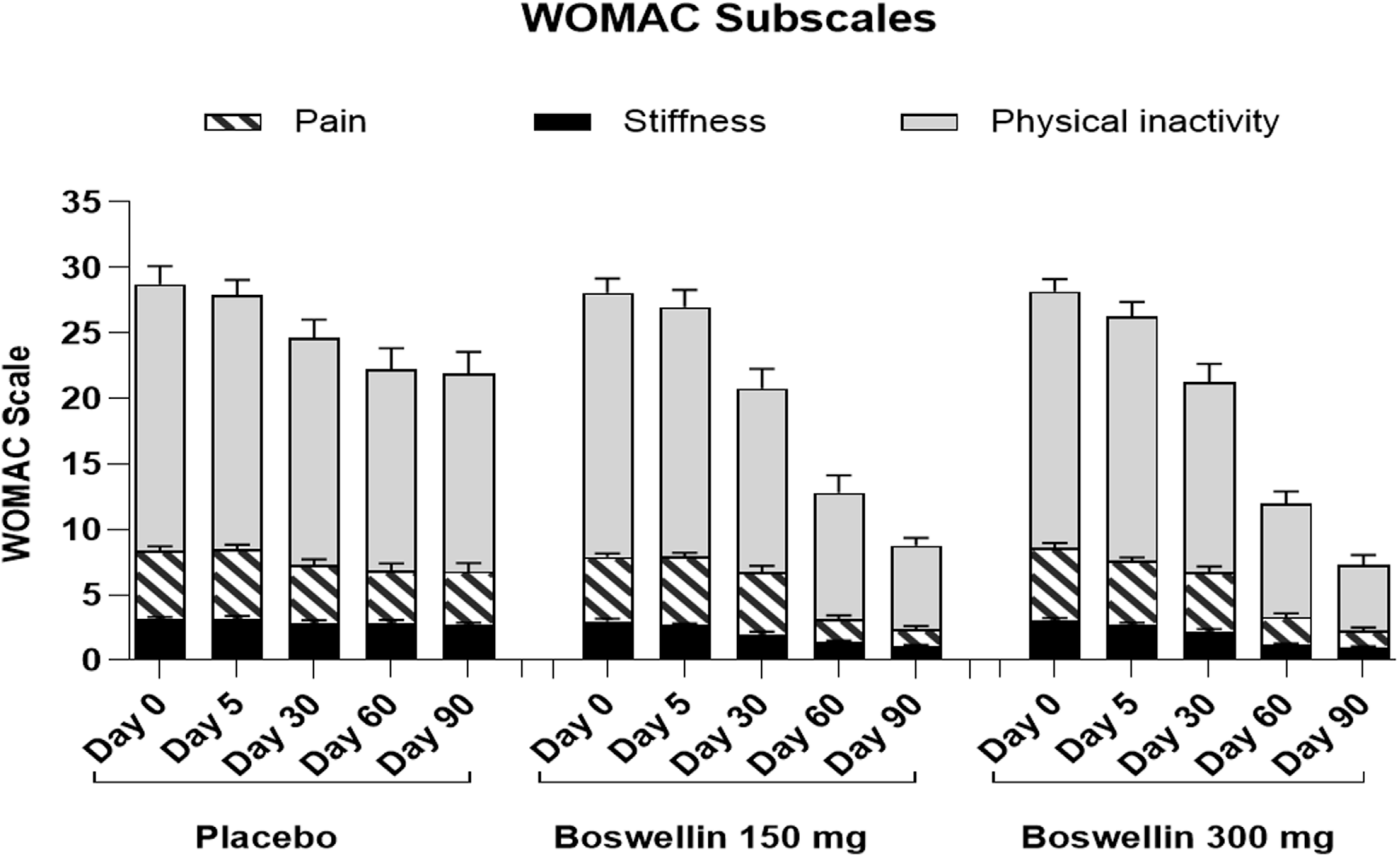
WOMAC pain, stiffness and difficulty in physical activity scores WOMAC pain, stiffness and difficulty in physical activity scores of placebo, BSE-150 and BSE-300 groups at different time points day 0, day 30, day 60 and day 90. Each bar represents Mean ± SEM. In comparison with Day 0, the mean scores in the treatment groups were tested for significance using Dunnett’s multiple comparisons test. The decrease in WOMAC pain and physical activity scores were significant (*p* < 0.05) on days 60 and 90 in comparison to day 0 in placebo, and from day 30 to 90 compared to day 0 (*p* < 0.001) in BSE-150 and BSE-300 compared to day 0. The stiffness improvement was significant only in BSE-150 and BSE-300 from day 30 onwards. The sub scores showed significant (*p* < 0.05) in BSE-300 on day 5 compared to day 0.

WOMAC total scores showed a gradual significant decrease in all three groups. In placebo the scores were (40.2 ± 10.4,39.5 ± 9.5, 34.7 ± 11.2, 31.9 ± 13.1 and 31.3 ± 13.2) on days 0,5, 30, 60 and 90, and in BSE-150 (38.9 ± 8.2, 37.6 ± 9.2, 29.5 ± 11.1,17.2 ± 9.2 and 12.1 ± 4.6) and in BSE-300 (39.8 ± 7.9 37.6 ± 9.2, 29.3 ± 11.4,15.9 ± 7.4 and 10.4 ± 5.7) on days 5, 30, 60, and 90. The decrease was highly significant in all the groups from days 30–90, compared to day 0, while the change was significant at day 5 also in the BSE-300 group (Figure 2). The interaction between groups and time was significant and the difference between placebo and BSE-150 and BSE-300 was significant at days 60 and 90 (*p* < 0.0001 at both time points) (Table 2). The three domains of WOMAC total score *viz.* pain, stiffness, and difficulty in physical activity subscales also followed a similar trend during the treatment period (Figure 4). Stiffness and physical activity sub-scores were found significantly lower (*p* < 0.0001) in BSE-150 and BSE-300 groups compared to placebo on day 30, day 60, and 90 (Figure 2) while the pain sub-score showed significant reduction (*p* < 0.0001) on day 60 and day 90 (Table 5). In a gender and age-specific analysis, an improvement in WOMAC total scores was observed at 60 days of supplementation in both males and females and in both age groups (<55 years and >55 years), as shown in Figure 3 and Table 3.

### 3.3 Effect of BSE Physician Global Assessment

On a physician assessment scale of 1–10, where a lower score indicates poor prognosis, significant improvements were observed in the BS-150 and BSE-300 groups after 30, 60, and 90 days of treatment. The score slightly increased from 4.9 ± 0.6 to 4.6 ± 0.7, 4.8 ± 0.7 4.6 ± 0.9 and 5.06 ± 0.9 (P=NS) on days 5, 30, 60 and 90 in placebo. The increase in BSE-150 was from 4.8 ± 0.5 to 4.6 ± 0.5, 5.2 ± 0.7, 5.9 ± 1.1, 6.8 ± 0.7 and from 4.8 ± 0.5, 4.7 ± 0.8, 5.3 ± 0.8, 6.1 ± 1.2(*p* < 0.001), 7.4 ± 1.07 in BSE-300 (*p* < 0.001) on days 5, 30, 60 and 90 respectively (Table 4). The change was significantly better (*p* < 0.001) than placebo in both the BSE groups compared to placebo on days 60 and 90 (Table 5).

**TABLE 4:** One-way repeated measure ANOVA analysis within the group with time.

Parameter	Day 0	Day 5	Day 30	Day 60	Day 90	*p*-value^a^
Physicians Global Assessment						
Placebo	4.94 ± 0.67	4.63 ± 0.76	4.78 ± 0.75	4.63 ± 0.91	5.06 ± 0.98	0.07
*p*-value^b^		0.14	0.79	0.32	0.91	
BSE-150	4.88 ± 0.49	4.59 ± 0.56	5.19 ± 0.74	5.97 ± 1.06	6.78 ± 0.71	<0.001
*p*-value^b^		0.08	0.03	<0.001	<0.001	
BSE-300	4.84 ± 0.51	4.74 ± 0.86	5.31 ± 0.82	6.16 ± 1.24	7.41 ± 1.07	<0.001
*p*-value^b^		0.51	0.005	<0.001	<0.001	
EQ5D index						
Placebo	0.51 ± 0.06	0.53 ± 0.06	0.58 ± 0.07	0.58 ± 0.08	0.61 ± 0.16	0.005
*p*-value^b^		0.44	0.001	0.003	0.003	
BSE-150	0.52 ± 0.04	0.54 ± 0.08	0.64 ± 0.09	0.71 ± 0.06	0.75 ± 0.10	<0.001
*p*-value^b^		0.51	<0.001	<0.001	<0.001	
BSE-300	0.55 ± 0.04	0.55 ± 0.11	0.64 ± 0.11	0.73 ± 0.07	0.79 ± 0.09	<0.001
*p*-value^b^		0.94	<0.001	<0.001	<0.001	
EQ5D Health score						
Placebo	41.06 ± 1.08	43 ± 1.32	46.38 ± 1.32	48.88 ± 1.46	52.81 ± 1.97	<0.001
*p*-value^b^		0.21	<0.001	<0.001	<0.001	
BSE-150	38.78 ± 1.25	43 ± 1.22	47.34 ± 1.65	56.75 ± 1.77	60.88 ± 2.30	<0.001
*p*-value^b^		0.005	<0.001	<0.001	<0.001	
BSE-300	39.35 ± 1.03	43.24 ± 1.1	46.91 ± 1.69	57.65 ± 1.32	64.03 ± 2.21	<0.001
*p*-value^b^		0.002	<0.001	<0.001	<0.001	
Number of laps						
Placebo	4.969 ± 0.23	5.094 ± 0.22	5.031 ± 0.22	4.969 ± 0.22	5.281 ± 0.21	0.026
*p*-value^b^		0.52	0.94	0.99	0.052	
BSE-150	4.813 ± 0.15	5.219 ± 0.16	5.344 ± 0.15	5.438 ± 0.14	5.875 ± 0.11	<0.001
*p*-value^b^		0.010	<0.001	<0.001	<0.001	
BSE-300	4.676 ± 0.21	4.941 ± 0.2	5.265 ± 0.17	5.441 ± 0.16	5.765 ± 0.10	<0.001
*p*-value^b^		0.1155	0.002	0.002	<0.001	
Total distance walked in 6 min						
Placebo	322.8 ± 13.4	321.3 ± 12.42	317.8 ± 11.89	315.6 ± 11.2	330 ± 11.15	0.18
*p*-value^b^		0.99	0.82	0.59	0.69	
BSE-150	309.4 ± 8.72	331.3 ± 9.3	341.3 ± 8.18	343.4 ± 7.28	375 ± 5.26	<0.001
*p*-value^b^		0.004	<0.001	<0.001	<0.001	
BSE-300	298.2 ± 11.22	309.9 ± 11.25	332.9 ± 9.86	345.3 ± 8.69	363.8 ± 6.31	<0.001
*p*-value^b^		0.1266	<0.001	<0.001	<0.001	

Mean ± Standard deviations are represented. One-way repeated measure analysis of variance (RMANOVA) was computed for each group at different time points.

^a^ RMANOVA.

^b^ Post hoc analysis using Dunnett’s multiple comparisons with Day 0.

**TABLE 5:** TWO-way ANOVA and comparison between groups (Secondary Endpoints).

Parameter	Mean (95% CI) of difference in mean between the groups										TWO way ANOVA		
	Day 0		Day 5		Day 30		Day 60		Day 90		Days	Group	Interaction
	Placebo vs BSE-150	Placebo vs BSE-300	Placebo vs BSE-150	Placebo vs BSE-300	Placebo vs BSE-150	Placebo vs BSE-300	Placebo vs BSE-150	Placebo vs BSE-300	Placebo vs BSE-150	Placebo vs BSE-300			
PGA	0.06 (−0.29–0.41)	−2.86 (−7.93 to 2.22)	−0.06 (−0.46 to 0.34)	−0.20 (−0.68 to 0.27)	−0.41 (−0.85to 0.04)	−3.75 (−9.36 to 1.86)	−1.34 (−1.94 to −0.75)	−3.23 (−6.24 to −0.22)	−1.72 (−2.23 to −1.20)	−2.79 (−3.88 to −1.69)	F (2, 95) = 3.69	F (1.21, 115.1) = 3.96	F (8, 380) = 1.71
*p*-value	0.905	0.362	0.925	0.567	0.082	0.243	<0.001	0.033	<0.001	<0.001	0.028	0.0412	0.094
WOMAC Pain	0.47 (−0.62–1.56)	−0.24 (−1.47 to 0.99)	0.56 (−0.57–1.70)	0.88 (−0.24–2.0)	0.56 (−0.93–2.06)	0.58 (−0.83–1.98)	3.69 (2.08–5.29)	3.52 (1.92–5.12)	4.44 (2.73–6.14)	4.55 (2.86–6.24)	F (2, 95) = 13.27	F (2.99, 284.5) = 167.4	F (8, 380) = 17.83
*p*-value	0.5568	0.8828	0.4654	0.1507	0.639	0.591	<0.0001	<0.0001	<0.0001	<0.0001	<0.0001	<0.0001	<0.0001
WOMAC Stiffness	0.13 (−0.47–0.72)	0.10 (−0.54–0.73)	0.47 (−0.24–1.18)	0.42 (−0.29–1.13)	0.84 (0.169–1.52)	0.67 (0.025–1.36)	1.47 (0.72–2.21)	1.67 (0.93–2.39)	1.63 (1.01–2.24)	1.72 (1.11–2.32)	F (2, 95) = 16.05	F (3.17, 300.8) = 58.21	F (8, 380) = 7.376
*p*-value	0.8706	0.9314	0.2603	0.3388	0.0107	0.061	<0.0001	<0.0001	<0.0001	<0.0001	<0.0001	<0.0001	<0.0001
WOMAC Function	0.66 (−3.63–4.94)	0.54 (−3.48–4.56)	0.88 (−3.38–5.14)	1.64 (−2.33–5.61)	3.84 (−1.16–8.85)	3.33 (−1.47–8.13)	9.47 (4.42–14.52)	10.28 (5.82–14.74)	13.16 (8.89–17.42)	14.64 (10.23–19.05)	F (2, 95) = 11.78	F (2.81, 266.5) = 189.1	F (8, 380) = 15.05
*p*-value	0.9282	0.9436	0.8749	0.5847	0.1638	0.2273	<0.0001	<0.0001	<0.0001	<0.0001	<0.0001	<0.0001	<0.0001
LFI (Severity)	0.06 (−0.67–0.79)	−0.18 (−0.93 to 0.57)	0.89 (0.071–1.71)	0.35 (−0.49–1.19)	1.33 (0.38–2.27)	1.00 (−0.160–2.15)	2.27 (1.25–3.28)	1.92 (0.98–2.88)	2.39 (1.46–3.32)	2.51 (1.59–3.43)	F (2, 95) = 12.89	F (3.05, 289.9) = 115.6	F (8, 380) = 9.991
*p*-value	0.977	0.830	0.030	0.57	0.003	0.104	<0.001	<0.001	<0.001	<0.001	<0.0001	<0.0001	<0.0001
LFI pain	−0.03 (−0.54 to 0.48)	−0.16 (−0.73 to 0.41)	0.66 (0.010–1.32)	0.29 (−0.35–0.93)	0.94 (0.34–1.53)	0.51 (−0.16–1.18)	1.19 (0.53–1.84)	1.03 (0.44–1.62)	1.38 (0.78–1.96)	1.40 (0.78–2.01)	F (2, 95) = 12.57	F (3.63, 345.3) = 56.35	F (8, 380) = 5.752
*p*-value	0.9883	0.7776	0.0524	0.5214	0.001	0.1735	0.0002	0.0003	<0.0001	<0.0001	<0.0001	<0.0001	<0.0001
LFI Daily living	0.00 (−0.61 to 0.61)	−0.03 (−0.69 to 0.63)	0.27 (−0.27–0.80)	−0.03 (−0.59 to 0.52)	0.36 −0.18–0.89)	0.33 (−0.28–0.95)	0.98 (0.44–1.52)	0.92 (0.39–1.44)	0.80 (0.233–1.36)	0.96 (0.42–1.50)	F (2, 95) = 4.470	F (3.05, 290.1) = 60.13	F (8, 380) = 5.091
*p*-value	>0.9999	0.9944	0.4648	0.9876	0.2529	0.3984	0.0001	0.0003	0.0036	0.0003	0.0140	<0.0001	<0.0001
EQ5D index	−0.01 (−0.04 to 0.02)	−0.03 (−0.06 to 0.001)	−0.01 (-0.05 to 0.034)	−0.02 (−0.07 to 0.03)	−0.06 (−0.11 to −0.004)	−0.06 (−0.11 to −0.002)	−0.13 (−0.17 to −0.08)	−0.15 (−0.19 to −0.10)	−0.15 (−0.23 to −0.07)	−0.19 (−0.26 to −0.11)	F (2, 95) = 23.93	F (2.83, 269.5) = 113.4	F (8, 380) = 8.814
*p*-value	0.8084	0.0401	0.8757	0.5779	0.0312	0.038	<0.0001	<0.0001	<0.0001	<0.0001	<0.0001	<0.0001	<0.0001
EQ5D Health Score	2.28 (−1.69–6.26)	−2.17 (−5.87 to 1.53)	0.00 (−4.31 to 4.31)	−0.24 (−4.34 to 3.87	−0.97 (−6.04 to 4.11)	0.64 −4.46–5.74)	−7.88 −13.40 to −2.35)	−8.77 (−13.51 to −4.04)	−8.06 −15.35 to −0.78)	−11.22 (−18.33 to −4.10)	F (2, 95) = 5.131	F (2.49, 236.7) = 96.53	F (8, 380) = 5.067
*p*-value	0.359	0.343	0.99	0.989	0.890	0.951	0.003	0.0001	0.026	0.001	0.0077	<0.0001	<0.0001
No. of laps	0.06 (−0.67–0.79)	−0.18 (−0.93 to 0.56)	−0.13 (−0.77 to 0.52)	0.15 (−0.55–0.86)	1.33 0.38–2.27)	1.00 (−0.160–2.15)	2.27 (1.25–3.28)	1.92 (0.97–2.88)	2.39 (1.46–3.32)	2.51 (1.59–3.43)	F (2, 95) = 19.55	F (2.82, 268.0) = 68.18	F (8, 380) = 8.615
*p*-value	0.977	0.830	0.888	0.862	0.003	0.104	<0.0001	<0.0001	<0.0001	<0.0001	<0.0001	<0.0001	<0.0001
Total distance walked	13.44 (−25.11–51.99)	24.64 (−17.35–66.62)	−10.00 (−47.32 to 27.32)	11.37 (−28.85 to 51.59)	−23.44 (−58.21 to 11.33)	−15.13 (−52.24 to 21.99)	−27.81 (−60.01 to 4.38)	−29.67 (−63.74 to 4.39)	−45.00 (−74.90 to −15.10)	−33.82 (−64.78 to −2.86)	F (2, 95) = 1.052	F (3.16, 300.7) = 36.90	F (8, 380) = 8.237
*p*-value	0.679	0.342	0.796	0.776	0.244	0.592	0.1031	0.099	0.002	0.029	0.3533	<0.0001	<0.0001

The difference in Mean value between the groups and the 95% of the difference are presented. Two-way ANOVA, was computed with groups and time as factors. Post hoc analysis was carried out by Turkey’s multiple comparisons between placebo and BSE-150, and BSE-300, and the *p* values are given for this comparison at different days. F distribution (the distribution of the ratio of two estimates of variance), degrees of freedom numerator (dfn) and degrees of freedom denominator (dfd) and the Two-way ANOVA *p* values for days, group and their interactions are represented in the last three columns. *p* < 0.05 was considered significant. PGA: physicians’ global assessment, WOMAC: Western Ontario McMaster Index, LFI: lequesne functional index.

### 3.4 Effect of BSE on walking

The ability to walk showed a significant improvement in individuals taking BSE-150 and BSE -300 from day 0 to day 90. The mean distance walked improved gradually from 309.4 ± 8.7m to 375 ± 5.2 m in the BSE-150 group, 298.2 ± 11.2m to 363.8 ± 6.3 m in the BSE-300 groups, and 322.8 ± 13.4 m to 330 ± 11.1 m in placebo in 90 days. The improvement was significant from day 5 onwards in BSE-150, day 30 onwards in BSE-300 (Table 4), and between placebo and BSE-150 and BSE-300 by day 90 (*p* < 0.0001) (Table 5). The mean change in the number of laps was also found significant in BSE-treated groups on days 60 and 90 compared with the placebo (Tables 4, 5).

### 3.5 Effect of BSE supplements on european quality of life 5 dimension (EQ(5D)) scores

The health-related quality of life, The European Quality of Life Index (EQ-5D), showed a marked improvement in BSE-150 (0.52 ± 0.04 to 0.75 ± 0.10, *p* < 0.001) and BSE 300 (0.55 ± 0.04 to 0.79 ± 0.09, *p* < 0.001) group as early as 30 days from the start of treatment. An improvement was observed in placebo also (0.51 ± 0.06 to 0.61 ± 0.16, *p* = 0.005). The improvement in quality-of-life index was significant compared to placebo in both the BSE treated groups (*p* = 0.03 at day 30 and *p* < 0.001 on days 60 and 90). The EQ(5D) health score showed significant improvements from day 5 onwards in BSE-150 and BSE-300. The score increased from 41.1 ± 1.1 to 52.8 ± 1.9 in placebo, 38.7 ± 1.2 to 60.8 ± 2.3 in BSE-150, and 39.3 ± 1.0 to 64.03 ± 2.2 in BSE-300. The change in health score was significantly better in BSE groups compared to placebo at 60 and 90 days (Table 4 & 5).

### 3.6 Effect of BSE supplements on lequesne functional index

**FIGURE 5:**
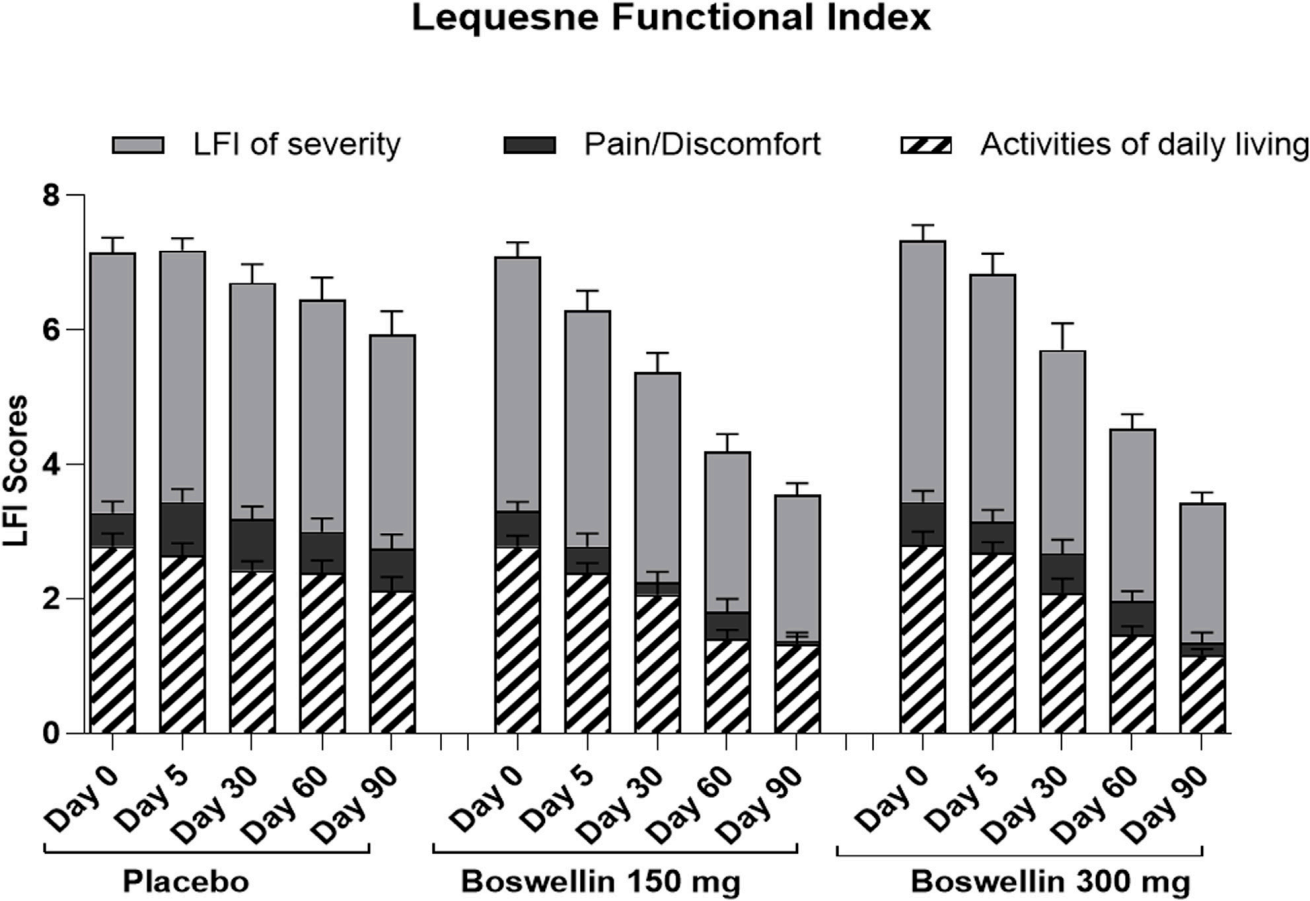
LFI pain, severity, and daily activity scores LFI pain, severity and daily activity scores of placebo, BSE-150 and BSE-300 groups at different time points day 0, day 30, day 60 and day 90. Each bar represents Mean ± SEM. In comparison with Day 0, the mean scores in the treatment groups were tested for significance using Dunnett’s multiple comparisons test. The decrease in LFI pain and daily activity and severity scores were significant (*p* < 0.05) on days 90 in comparison to day 0 in placebo, and from day 30 to 90 compared to day 0 (*p* < 0.001) in BSE-150 and BSE-300 compared to day 0. The pain and severity improvement was significant (*p* < 0.05) in BSE-150 on day 5 compared to day 0.

The Lequesne functional index is specific for knee OA and is recorded in terms of pain, severity, and activities related to daily living. Significant improvements (*p* < 0.01) were recorded in both BSE-150 and BSE-300 groups on days 30, 60, and 90 compared to day 0. The pain and severity showed a significant improvement in BSE-150 as early as day 5 (Figure 5). These improvements in BSE-150 and BSE-300 groups were significantly better (*p* < 0.001) compared to Placebo especially from day 60 onwards (Table 5).

### 3.7 Effect of BSE supplements on inflammatory markers

The mean (95% CI) Hs CRP levels increased in placebo from 6.46 (3.64, 9.29) to 8.45 (6.69, 10.21) in placebo and showed a mild decrease in the BSE-150 [9.29 (5.89,12.68) to 8.34 (6.22,10.48)] and BSE-300 [11.90 (8.25,15.55) to 8.76 (5.87,11.66) pg/mL, *p* = 0.018]. The serum levels of TNF- α did not change in placebo [56.88, (16.79, 96.96) to 57.94 (28.61,87.27)] pg/mL and decreased from 38.05 (10.02,66.08) to 20.47 (8.86,32.08) pg/mL in BSE-150 and 40.15 (18.58,61.72) to 31.64 (5.64,57.65) pg/mL in BSE-300. The concentrations were highly variable in individuals and the change in the concentrations were not statistically significant. Similarly, the serum levels of IL6 were highly variable and were not statistically significant between the groups (Table 6).

**TABLE 6:** Comparison of inflammatory marker levels from the baseline.

Inflammatory markers	Day 0	Day 90	*p*-value^a^	*p*-value^b^
hs-CRP				
Placebo	6.46 (3.64, 9.29)	8.45 (6.69, 10.21)	0.008	
Boswellin (150 mg)	9.29 (5.89,12.68)	8.34 (6.22,10.48)	NS	NS
Boswellin (300 mg)	11.90 (8.25,15.55)	8.76 (5.87,11.66)	0.018	
TNF -Alpha				
Placebo	56.88 (16.79, 96.96)	57.94 28.61,87.27)	NS	
Boswellin (150 mg)	38.05 (10.02,66.08)	20.47 (8.86,32.08)	NS	NS
Boswellin (300 mg)	40.15 (18.58,61.72)	31.64 (5.64,57.65)	NS	
IL-6				
Placebo	30.86 (10.03, 51.68)	52.16 (24.96,79.36)	0.001	
Boswellin (150 mg)	50.67 (31.98,69.35)	45.50 (24.82,66.18)	NS	NS
Boswellin (300 mg)	43.94 (19.32,68.56)	29.71 (10.51,48.91)	0.015	

Mean and 95% Confidence interval of mean are given in the table, since the variation within groups was very high. hs-CRP- High sensitivity C reactive protein, TNF-α- Tumor necrosis factor, IL-6- Interleukin −6.

*p* value^a^ represents the difference between day 0 and day 90 within the group. *p* value^b^ represents the difference between the three groups in the change in biomarker levels.

### 3.8 Effect of BSE treatment on radiological X-ray examination

**FIGURE 6:**
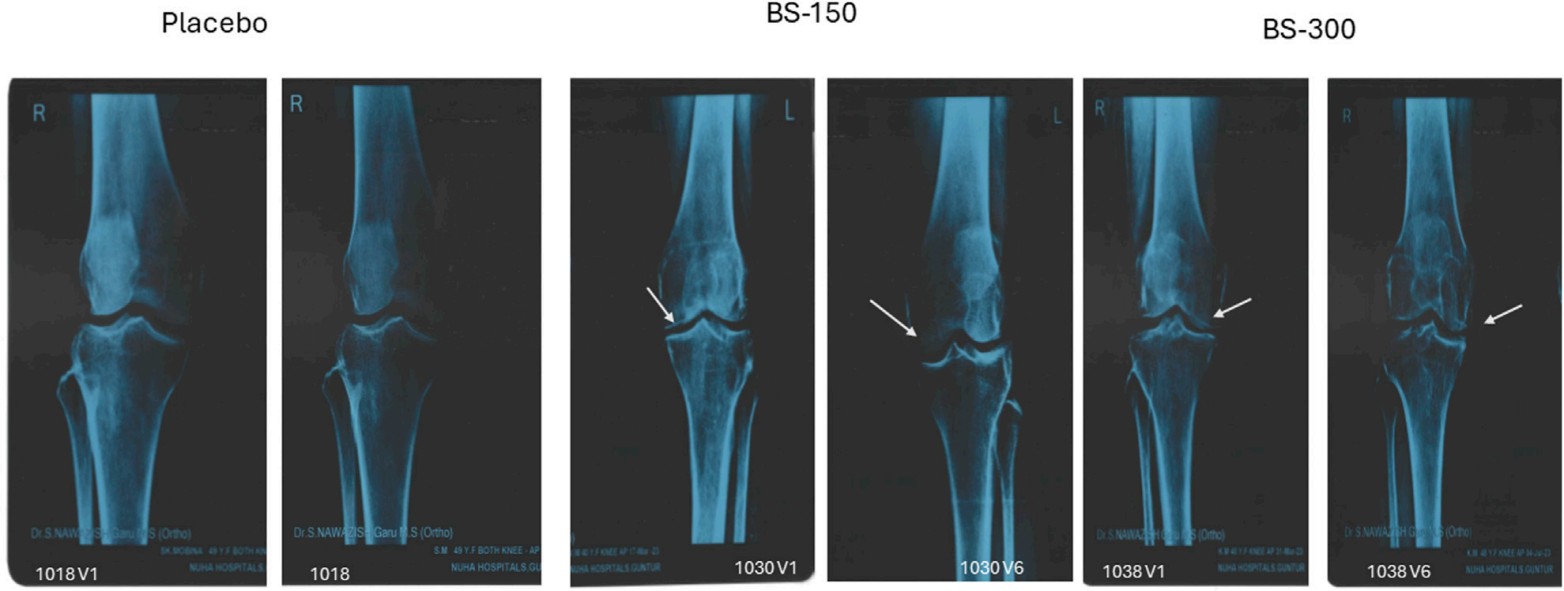
Radiological X-ray image. Radiological X-ray images of the knee of participants captured on screening and end of the study. Significant improvements in osteoarthritis was observed in the knee joint. The gap between the knee joints (white arrow) increased significantly in BSE-150 and BSE-300 supplemented participants.

Examination of radiological X-ray images revealed narrowing of joint space with osteophytes in the knee of the participants in all the groups at screening. BSE-150 and BSE-300 groups showed considerable improvements in 90 days. The gap between the bones in the knee joints improved with a reduction in osteophytes in 13.3% of participants in placebo, 41.9% of participants in BSE-150, and 44.11% in BSE-300 groups (Figure 6)

### 3.9 Safety parameters

All the patients who completed the study were analyzed for the safety of the BSE supplement. Mild adverse events were reported by three participants, one from the placebo group and two from the BSE-300 group. The participant in the placebo group reported mild body pain which got resolved in a day. One participant in BSE-300 reported a headache and gastric problem, which was mild and resolved without any medication within 24 h. The second participant in BSE-300 developed elevated blood glucose and was prescribed metformin 500 mg, which was considered not related to the supplement by the physician (Table 7). The clinical laboratory parameters were at the normal level for all the patients (Supplementary Tables S1-3).

**TABLE 7:** Safety Data- Adverse events.

Group	AE	Start date and time	Stop date and time	Outcome	Severity	Possible relationship to study drug	Withdrawn due to AE?
Placebo	Body Pain	17–03-23	18–03-23	Resolved	Mild	No	No
BSE-300	Headache, Gastric problem	29–04-23	30–04-23	Resolved	Mild	No	No
BSE-300	Elevated Blood Glucose	23–09-23	Ongoing	Resolved with Sequelae	Mild	No	No

Description of mild adverse effects experienced by participants during the study.

## 4 Discussion

In this randomized, placebo-controlled clinical study, dietary supplementation with Boswellin^®^ Super (BSE) standardized for 30% AKBBA, and 50%–55% beta boswellic acids at two doses was observed to improve joint health, and mobility in adults with newly diagnosed degenerative osteoarthritis. BSE supplementation was associated with a significant improvement in quality of life, with statistically significant decreases in pain scores, and WOMAC total and sub-scale scores. The Lequesne functional index, ability to walk and functional activities significantly improved in the individuals consuming BSE. The improvements were significant for pain and stiffness as early as 5 days after starting the supplement. Interestingly the benefits of pain reduction were observed 15 days after stopping the supplements in the BSE groups. None of the participants required any rescue medication, probably because we had enrolled individuals with moderate disease.

Boswellia extracts containing boswellic acids as the major pharmacologically active compound have been clinically proven to improve pain, physical function, and inflammation in arthritic patients (Kimmatkar et al., 2003; Gupta et al., 2011; Majeed et al., 2019). A recent meta-analysis including data from seven studies involving 545 patients, concluded that Boswellia extract may be an effective and safe treatment option for OA patients (Yu et al., 2020). Symptomatic improvements were reported with doses up to 6 g of *B. serrata*, of undetermined composition of β-boswellic acids (Sontakke et al., 2007; Gupta et al., 2011, compared the efficacy of *B. Serrata* (1g/day) with valdecoxib in a 6-month trial and showed that the onset of action for Boswellia extract was slower but its effect persisted even after stopping therapy while valdecoxib showed faster effects, which diminished rapidly after stopping the treatment (Sontakke et al., 2007). An extract of *B. Serrata* containing 40% boswellic acids, supplemented at 1 g/day was reported to reduce pain and joint stiffness in OA patients (Kimmatkar et al., 2003). Enriched extracts of *B serrata* could reduce pain and improve physical functioning significantly at 100 and 250 mg/day in OA patients (Sengupta et al., 2008; Sengupta et al., 2010; Vishal et al., 2011). Our earlier study with Boswellin^®^ Super at 340 mg/day showed significant improvements in physical functions compared to placebo in newly diagnosed or untreated patients with OA of the knee (Majeed et al., 2019).

Our results agree with the earlier studies and reiterate the safety of a BSE-300 twice-a-day (600 mg/day) dose of a standardized BSE (30% AKBBA, and 50%–55% total beta boswellic acids) for 90 days in arthritis patients.

The effect of Boswellin^®^ Super supplementation was evident as early as 5 days after the start of the study, resulting in VAS reduction by 7.4% and 14.3% at BSE-150 and BSE-300 groups (300 and 600 mg/day) doses respectively compared to baseline. By 90 days this improved to 45.3% and 61.9% and the effect was sustained for 15 days after cessation of the supplement (43.6% and 55.7% respectively). The WOMAC- total score improved by 68.5% and 73.6% in BSE-150 and BSE-300 doses respectively compared to 22% in placebo by 90 days. Similar significant improvements were observed in WOMAC sub scores, Lequesne functional index, EuroQol index and health score, and the ability to walk with both doses of Boswellin, which were also significantly better than the placebo. There was no significant difference between the two doses of Boswellin^®^ Super, suggesting that BSE-150 twice a day (300 mg/day) was sufficient to reduce the pain, stiffness, and functional activities due to osteoarthritis. The placebo group did show some reduction in VAS and WOMAC scores, which could be attributed to the placebo effect (Turner et al., 1994; Walach et al., 2005). Gender and age-specific analysis revealed an improvement in VAS pain and WOMAC total scores in both men and women and both 40–55 years and 55–70-year-old participants.

Joint inflammation triggered by inflammatory mediators and cytokines is the primary mediator of osteoarthritis (Goldring and Otero, 2011). As a consequence, inflammatory cytokines like IL-1β, IL-6, and TNF-α are higher in the blood, synovial fluid, and cartilage tissue of patients with OA, and can trigger matrix degradation and increase the expression of nitric oxide, and PGE2 in chondrocytes (Wojdasiewicz et al., 2014). In our earlier study in a preclinical model, we demonstrated that BSE with the same composition as used in this study, reduces inflammation, and oxidative stress and, most importantly, preserves the matrix proteins by inhibiting the enzymes that hydrolyze The ECM proteins in animal models of arthritis (Majeed et al., 2021). BSE could inhibit the phosphorylation of NF-κB (p65), inducible nitric oxide levels, and quench intracellular ROS in macrophages in the *in vitro* studies, confirming its anti-inflammatory and antioxidant activity.

Although we observed a reduction in serum markers like TNF-α, IL6, and hs-CRP in the BSE-supplemented participants, we observed wide variation in the levels and hence the reduction was not statistically significant compared to placebo. Radiological examination of patients consuming BSE showed improvements in the space between bones in the knee joint, and reduction in osteophytes, suggesting its benefit in knee OA. There were no serious adverse events reported during the study at both doses, reiterating the safety of BSE for human consumption. The preclinical safety of this product was established by acute, chronic, and genotoxicity studies as per regulatory requirements (Majeed et al., 2020b).

The study was conducted in multiple sites at two different dosages with multiple arthritis-specific questionnaires, which can be termed as the strength of the study. Although the study included 105 participants, the numbers in each group were relatively lower. Larger cohort studies would help in reducing the variability in biomarkers and help in establishing a mechanism of action for the supplement, which is a limitation of the study. Larger participant size would also allow a meaningful subgroup analysis by gender and age group. Although we did a subgroup analysis, the number of samples in each subgroup was small, and the results may not be very robust. The study was conducted in the Southern part of India, limiting the ethnicity of the participants and in individuals with newly diagnosed degenerative hypertrophy OA, which limits the generalization of the results to severe arthritis. However, the study establishes the benefit of standardized Boswellia extract in improving joint health and mobility. The benefits persisted for 15 days after the supplements were stopped, and they were noticeable as early as 5 days after the doses were started. Future studies for a longer duration in multi-ethnic population with different severity of the disease and in individuals taking concomitant medication would be helpful in positioning the extract as a supplement for managing joint health in osteoarthritis.

## 5 Conclusion

The results from the study provide clinical evidence that **Boswellin® Super**, a standardized extract from *B serrata* containing 30% AKBBA and 50%–55% total beta boswellic acids can be used as a safe and effective supplement to support joint health and mobility in the management of osteoarthritis.

## Data Availability

The original contributions presented in the study are included in the article/Supplementary Material, further inquiries can be directed to the corresponding author.
