# Supplementary material for: A standardized Boswellia serrata extract shows improvements in knee osteoarthritis within five days-a double-blind, randomized, three-arm, parallel-group, multi-center, placebo-controlled trial
Source: Front Pharmacol. 2024 Jul 18;15:1428440. doi: 10.3389/fphar.2024.1428440 (PMC11291344; doi:10.3389/fphar.2024.1428440)
Supplement: Supplementary file 1 [file DataSheet1.pdf]

# *Supplementary Material*

## 1 Supplementary Data

### 1.1 Sample Size Determination:

We used research publication referenced below. As per research publication, baseline values of Western Ontario and McMaster Universities Arthritis Index (WOMAC) total score measured at baseline were:

Group 1 (n=61) baseline-Total WOMAC Score mean=55.8, SD=13.0

Group 2 (n=66) baseline-Total WOMAC Score mean=54.1, SD=13.9

We chose mean=55 and SD=13.5 hours as being towards the center of the two baseline results.

An SD=13.5 with mean=55 means the SD is about 0.2 times (base correlation) the size of the mean. That is, the coefficient of variation (CV) is 0. The mean Total WOMAC Score in the study is 55 and we assume the standard deviation is 13.5.

Using these inputs with 80% power and alpha=0.05 significance level assuming correlation of 0.2, the required total sample size is 90 for evaluation.

Allowing for 15% drop-out rate, the required sample size for recruitment is total of 105 in 1:1:1 ratio between three active (*Boswellia serrata* extract 175 mg b.i.d, *Boswellia serrata* extract 350 mg b.i.d and Celecoxib 100 mg OD) treatment groups (i.e. 35 per treatment group).

fixed

## 2. Study population

### 2.1. Inclusion Criteria

Participants of either sex, 40 to 75 years of age.

Participants newly diagnosed with degenerative hypertrophy OA (imaging diagnosis)

Participants whose knee KL (Kellgren-Lawrence) grade is I-II.

Participant with the pain perception ranging from 30 to 100 when measuring VAS (Visual Analog Scale) of 0 – 100mm.

Willing to come for regular follow -up visits.

Able to give written informed consent and comply with the requirements of the trial.

**2.2 Exclusion Criteria**

Known history of hypersensitivity to herbal extracts or dietary supplements.

Pregnant women, lactating women and women of childbearing potential not following adequate contraceptive measure, women who were found positive for urine pregnancy test.

Non degenerative joint diseases or other joint degenerative diseases (musculoskeletal disorders) which will interfere with the evaluation of OA (Rheumatoid arthritis, active gout, recent joint trauma (target joint), or joint infection).

Participant with moderate or severe synovitis: KL (Kellgren-Lawrence) grade III or higher.

Participants incapacitated or bound to wheelchair or bed and unable to carry out self-care activities.

Participant with a history of knee or hip replacement surgery.

Treatment with intra-articular injection of corticosteroids into the knee within 3 months preceding study.

Pre-existing or recent onset of demyelinating disorders or participants with a ruptured meniscus.

Evidence of several renal, hepatic, or hemopoietic diseases or severe cardiac insufficiency as revealed by laboratory investigations.

Participant with chronic diseases of kidney, liver, or gastrointestinal tract, cardiovascular, endocrine, or nervous system.

Participants with uncontrolled hypertension and uncontrolled diabetes, clinically significant untreated hyperlipidemia in context of a cardiovascular risk.

Participants who have used the following substance within 1 week prior to screening – glucosamine sulfate, chondroitin sulfate, NSAID, glucocorticoids, or steroids.
Participant who has used hyaluronate, any dietary supplement within a month prior to screening.

History of having received any investigational drug or participated in any other clinical trial which ended in the preceding month or currently ongoing.
Alcoholics (inability to control drinking due to both physical and emotional dependence on alcohol characterized by uncontrolled drinking and preoccupation with alcohol), History of chronic smoking (more than 2 cigarettes a day) and drug addicts.

Ayurvedic formulation or any form of CAM (Complimentary Alternative Medicine) therapy in the preceding 2 months.

Non-co-operative attitude of Participant.

Participant who has hip or back pain that interferes with the walking or movement tests.

Any condition that in the opinion of the investigator does not justify the Participant’s inclusion in the study.

## 2 Supplementary Figures and Tables

### Figure 1 HPLC chromatogram of *Boswellia serrata* extract

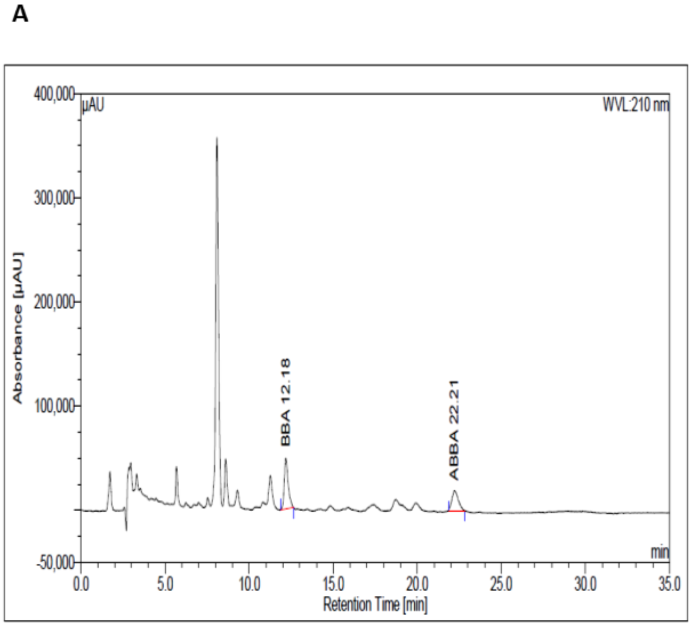


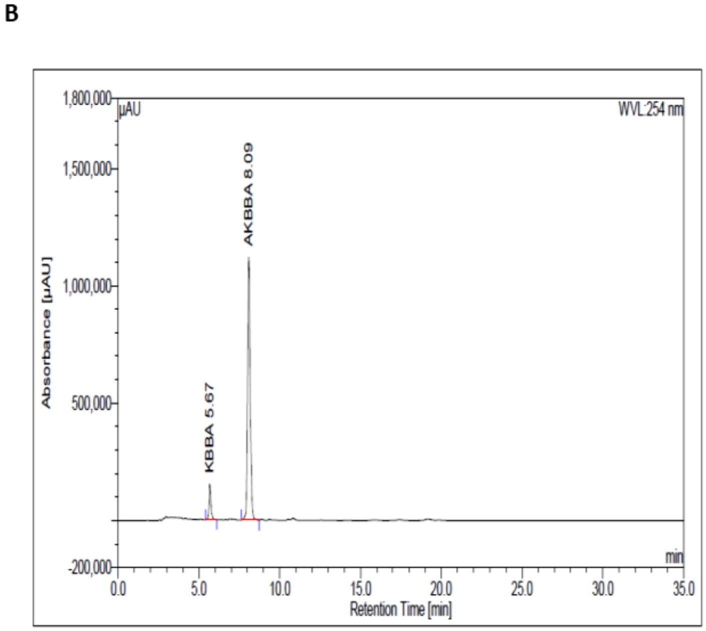

## 2.1 Safety Results

**Table 1: Hematological parameters**

| Parameter | Day 0 | | | Day 90 | | |
|---|---|---|---|---|---|---|
| | **Placebo** | **BSE-150** | **BSE-300** | **Placebo** | **BSE-150** | **BSE-300** |
| Haemoglobin (g/dl) | 12.27±2.19 | 13.21±1.71 | 12.38±2.12 | 12.17±2.04 | 15.8±14.18 | 12.56±2.04 |
| RBC (million/mm3) | 4.69±0.56 | 4.78±0.5 | 4.6±0.58 | 4.65±0.56 | 4.78±0.45 | 4.6±0.56 |
| Platelet Count (lakhs/mm3) | 3.03±0.79 | 7.6± 28.09 | 2.77±0.69 | 3.23±0.73 | 4.84±6.78 | 2.74±0.68 |
| Packed Cell Volume (%) | 35.79±5.95 | 37.88±4.21 | 35.92±5.71 | 34.84±5.34 | 36.99± 7.5 | 35.87±6.06 |
| Mean Cell Volume (FL) | 76.66±9.55 | 79.45±6.41 | 78.51±7.3 | 75.26±9.73 | 79.73±5.73 | 78.57±7.88 |
| Mean Platelet Volume | 10.49±1.42 | 10.69±1.33 | 10.48±2.27 | 10.43±1.39 | 10.72±1.24 | 10.88±1.42 |
| MCH (pg) | 26.3±3.88 | 27.6±3.02 | 28.12±6.27 | 26.17± 4 | 27.5±2.89 | 27.3±2.97 |
| MCHC (%) | 34.2±1.4 | 34.84±1.52 | 34.39±1.3 | 34.7±1.65 | 34.76±1.5 | 35.12±2.31 |
| Total Leukocyte Count (cells/mm3) | 7845.71± 1682.44 | 8086.86± 1944.13 | 7577.14± 2081.88 | 8214.9± 2208.63 | 8110.13± 1580.4 | 7714.71± 2044.54 |
| Lymphocytes (%) | 34.38±6.65 | 34.85±8.01 | 34.4±9.58 | 35.5±7.29 | 33.53±6.62 | 34.81± 5.83 |
| Eosinophils (%) | 3.48±1.65 | 4.97±4.98 | 2.69±1.7 | 2.8±1.65 | 4.18±2.78 | 2.86±1.52 |
| Monocytes (%) | 6.31±1.43 | 6.74±1.83 | 6.35±1.93 | 6.73±1.92 | 6.93±1.84 | 6.53±2.07 |
| Neutrophils (%) | 54.88±6.68 | 53.04±9.05 | 55.63±10.34 | 52.26±12.61 | 54.34±7.75 | 54.84± 6.06 |
| Basophils (%) | 0.65±0.5 | 0.63±0.46 | 0.71±0.55 | 2.48±9.94 | 0.76±0.55 | 0.76±0.53 |

All data presented as Mean± Standard deviation. None of the parameters were significantly different from baseline to end of the study.

**Table 2: Lipid parameters (mg/dL)**

| Parameter | Day 0 | | | Day 90 | | |
|---|---|---|---|---|---|---|
| | Placebo | BSE-150 | BSE-300 | Placebo | BSE-150 | BSE-300 |
| TC | 185.17±32.44 | 180.43±32.69 | 181.86±32.27 | 175.16±36.29 | 171.88±21.28 | 182±27.43 |
| HDL-C | 40.67±4.68 | 39.4±4.95 | 39.77± 3.8 | 39.94±3.19 | 38.78±3.82 | 40.44± 2.95 |
| LDL-C | 115.91± 28.83 | 107.42± 36.81 | 111.19± 34.66 | 110.44± 29.46 | 104.37± 19.44 | 110.99 ±23.7 |
| VLDL-C | 27.93±11.38 | 30.99±15.96 | 33.78±19.38 | 29.18±16.03 | 28.11±11.61 | 29.95±14.19 |
| TG | 147.8±81.64 | 154.94±79.81 | 154.14±65.56 | 137.61±38.75 | 141.03±57.78 | 149.88±70.89 |

Data presented as Mean± Standard deviation. None of the parameters were significantly different from baseline to end of the study. TC- Total Cholesterol, HDL-C- High density lipoprotein cholesterol, LDL- Low density lipoprotein cholesterol, VLDL- Very low-density lipoprotein cholesterol, TG- Triglycerides

**Table 3: Other Biochemical parameters**

| | Day 0 | | | Day 90 | | |
|---|---|---|---|---|---|---|
| | Placebo | BSE-150 | BSE-300 | Placebo | BSE-150 | BSE-300 |
| **FBS (mg/dl)** | 96.51±20.98 | 90.6±10.84 | 92.03±14.5 | 94.29±13.31 | 91.75± 5.3 | 93.97±21.68 |
| **Liver function** | | | | | | |
| Total Bilirubin (mg/dl) | 0.58±0.27 | 0.65±0.31 | 0.79±1.07 | 0.58±0.25 | 0.67±0.26 | 0.63±0.25 |
| Alkaline Phosphatase (IU/l) | 99.06±34.24 | 93.6±27.07 | 91.97±20.52 | 97.23±32.89 | 97.03±23.51 | 93.53±18.39 |

| | | | | | | |
|---|---|---|---|---|---|---|
| SGOT (U/l) | 31.06±9.08 | 30.37±9.19 | 28.2±7.38 | 28.19±5.8 | 28.22± 7.4 | 29.05± 12.6 |
| SGPT (U/l) | 31.8±15.26 | 30.71±12.17 | 35.66±30.11 | 25.91±7.26 | 29±7.39 | 29.38± 9.71 |
| **Renal Function** | | | | | | |
| Serum Creatinine (mg/dl) | 0.88±0.13 | 0.94±0.16 | 0.91±0.14 | 0.87±0.11 | 0.9±0.09 | 0.93± 0.15 |
| BUN (mg/dl) | 10.05±3.01 | 10.02±2.83 | 14.81±23.57 | 11.08±3.94 | 9.97±2.52 | 11.86± 3.26 |
| Urea (mg/dl) | 21.57 | 21.06±6.77 | 22.57±7.66 | 22.26±5.53 | 22.03± 5.4 | 25.47± 7.02 |
| Uric Acid (mg/dl) | 4.85±1.39 | 4.77±1.12 | 4.85±1.49 | 4.85±1.25 | 4.58±1.19 | 4.99± 1.28 |
| eGFR (ml/min) | 81.09±27.99 | 78.51±16.62 | 75.65±17.85 | 77.17±12.06 | 80.25±11.87 | 72.35±10.91 |
| Sodium (mmol/l) | 140.75±11.2 | 142.31±5.27 | 142.6±2.02 | 142±1.88 | 142.75±1.48 | 142.41±2.19 |
| Potassium (mmol/l) | 5.37±6.73 | 4.17±0.26 | 5.2±6.07 | 4.1±0.25 | 4.22±0.44 | 4.21±0.32 |
| Chloride (mmol/l) | 105.09±2.06 | 105.31±1.68 | 104.57±1.8 | 103.77±1.63 | 103.84±1.9 | 104.15±2.13 |
| Specific Gravity | 1.02±0.01 | 1.02±0.01 | 1.02±0.01 | 1.02±0.01 | 1.02±0 | 1.02±0.01 |
| pH | 5.6±0.53 | 5.61±0.64 | 5.56±0.51 | 5.41±0.48 | 5.37±0.39 | 5.39±0.41 |
| **Thyroid function** | | | | | | |
| T3 (ng/dl) | 1.46±0.25 | 1.5±0.28 | 1.45±0.32 | 1.47±0.26 | 1.62±0.24 | 1.4±0.31 |
| T4 (ng/dl) | 9.15±1.27 | 9.32±1 | 9.13±1.21 | 9.54±1.56 | 9.34±1.05 | 8.99±1.79 |
| TSH (ng/dl) | 2.77±1.13 | 2.97±1.95 | 2.48±1.23 | 2.81±1.07 | 3.12±1.86 | 3.07±1.55 |

**Research Publication:**

Chang, S., Song, Y. and Nah, S. (2018). The Clinical Efficacy and Safety of Gumiganghwal-Tang in Knee Osteoarthritis: A Phase II Randomized Double Blind Placebo Controlled Study. Evidence-Based Complementary and Alternative Medicine, 2018, pp.1-8.
